# Fluency, prediction and motivation: how processing dynamics, expectations and epistemic goals shape aesthetic judgements

**DOI:** 10.1098/rstb.2023.0326

**Published:** 2024-01-29

**Authors:** Jenny Yoo, Katarzyna Jasko, Piotr Winkielman

**Affiliations:** ^1^ Department of Psychology, University of California, 9500 Gilman Drive, La Jolla, CA 92093-0109, USA; ^2^ Jagiellonian University, Institute of Psychology, Ingardena 6, 30-060 Krakow, Poland; ^3^ SWPS University, Chodakowska 19/31, 03-815 Warsaw, Poland

**Keywords:** fluency, aesthetics, evaluation, predictive processing framework, epistemic motivation model

## Abstract

What psychological mechanisms underlie aesthetic judgements? An influential account known as the Hedonic Marking of Fluency, later developed into a Processing Fluency Theory of Aesthetic Pleasure, posits that ease of processing elicits positive feelings and thus enhances stimulus evaluations. However, the theory faces empirical and conceptual challenges. In this paper, we extend it by integrating insights from predictive processing frameworks (PPF) and the epistemic motivation model (EMM). We propose four extensions. First, fluency of a stimulus depends on perceivers' expectations—their internal model of the world. Second, perceivers also form expectations about fluency itself and thus can experience surprising fluency. These expectations can come from the individual's history, their current task and their environment. Third, perceivers can value fluency but also disfluency, reflecting their non-directional epistemic goals. Fourth, perceivers also have directional epistemic goals, preferring specific conclusions or belief content. Consequently, affective reactions depend on whether the stimulus satisfies those goals. These directional epistemic goals may override concerns about fluency or change the value of fluency associated with specific content. We review supporting evidence and introduce novel predictions. By integrating insights from PPF and EMM, our framework can better capture established fluency effects and highlights their limitations and extensions.

This article is part of the theme issue ‘Art, aesthetics and predictive processing: theoretical and empirical perspectives’.

## 1. Introduction

What determines people's reactions to art and design? Why do certain artworks, styles or designs endure whereas others fade into oblivion? Why are some pieces appealing despite their upsetting content, ambiguity, or even inscrutability? Why do viewers admire certain objects in museums that they ignore or even abhor in everyday life? These are only some of the many questions surrounding the puzzle of aesthetic judgement.

In fact, the very concept of ‘aesthetic judgement' is a puzzle. After all, interacting with a work of art involves an arch of mutually dependent perceptual, cognitive and affective processes that generate aesthetic experience [1,2]. Still, viewers do eventually express their judgements, and researchers in aesthetics do ask participants for their ratings. For instance, they may ask people to evaluate how beautiful, intriguing, valuable, innovative, interesting or artistic a given piece of art is. We define aesthetic judgements as an outcome of the mixture of cognitive and affective processes, similar to the proposal of Leder & Nadal [3]. The cognitive elements encompass recognition of basic elements of the artwork and beliefs about its meaning, the artist and the place of the artwork in art history and larger societal issues, as well as individual expectations and goals. The affective elements are feelings evoked by the art piece such as pleasure, beauty, wonder, boredom, doom or surprise. Intrinsic to this affective-cognitive dynamic is the fact that some aesthetic judgements are grounded in more low-level automatic processes, resulting in gut-level aesthetic pleasure, whereas others are grounded in more high-level cognitive elaboration [4]. Our current proposal is consistent with these multi-faceted, multi-level approaches to aesthetic experience and resulting aesthetic judgement.

Our contribution here focuses on one account of aesthetic experience and resulting judgements, which originates in a general cognitive theory of processing fluency [5]. The *Hedonic Marking of Fluency Account* was developed as an explanation for the role of fluency in evaluative judgements [6]. Shortly after, it was extended into an account of aesthetic judgements, and is known as the *Processing Fluency Theory of Aesthetic Pleasure* [7]. This theory has been influential in both the scientific and popular literature on aesthetics [8]. However, serious conceptual and empirical challenges have emerged since these ideas were proposed 20 years ago.

The goal of the current paper is to revise and update the hedonic fluency framework by incorporating insights from predictive processing frameworks (PPF) [9] and the epistemic motivation model (EMM) [10]. These two theoretical developments have important parallels [11,12]. Unfortunately, their joint application has not yet been attempted in the domain of aesthetic experience. We highlight the role of four factors that were either neglected or given minimal attention in the original Hedonic Fluency Theory and its application to art. The first two factors have to do with expectations and predictions and are directly connected to the PPF. The next two factors have to do with epistemic goals and come from the EMM.

**Figure 1.**
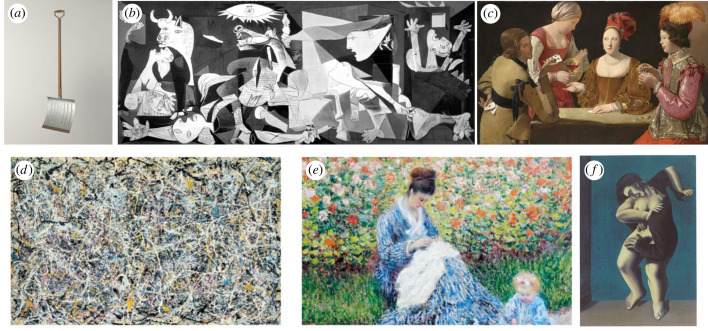
(*a*) Duchamp's ‘In Advance of a Broken Arm’, (*b*) Picasso's ‘Guernica’, (*c*) La Tour's ‘The Cheat with the Ace of Clubs’, (*d*) Pollock's ‘Number 1’, (*e*) Monet's ‘Camille Monet and a Child in the Artist's Garden at Argenteuil’, (*f*) Magritte's ‘Les Jours gigantesques’.

Here is a preview of our argument. First, people may differ in how much fluency they experience in reaction to the same stimulus, like the pieces shown in figure 1. These differences can depend on their prior expectations formed by personal history or the larger context. For example, take an everyday object, like a simple shovel (Duchamp's ‘In Advance of a Broken Arm’). When initially noted hanging in a museum, the shovel might be *less* fluent than in a garage. Similarly, the same shovel will feel *more* fluent for an art critic than for a casual visitor. Second, people have (explicit or implicit) expectations about the fluency itself, and thus may experience different degrees of ‘surprise’ in fluency. For example, a seemingly chaotic, impenetrable piece of modern art may be surprisingly easy on the eyes (e.g. Jackson Pollock's No. 1). Third, depending on non-directional epistemic goals of the perceiver (for non-specific certainty or uncertainty) fluency itself can be evaluated as good or bad, which will result in different respective aesthetic judgements. For instance, when encountering an ambiguous piece (e.g. Magritte's Les Jours Gigantesque), viewers may have a goal of quick and complete understanding, or they may prefer to be kept in a state of suspension or puzzlement. Fourth, what also matters for evaluation is the content of specific epistemic goals (goals related to belief content). For example, take a communist whose preferred conclusion is that a work of art favourably represents communism. This epistemic goal may lead to a positive response to a skillful artwork of social realism. Similarly, a viewer with a preference for content favourable to family values, will experience a positive reaction to a painting displaying a mother and child (e.g. Monet's family scene). Critically, these directional epistemic goals can change whether and how the fluency of specific content matters in final evaluations.

**Figure 2.**
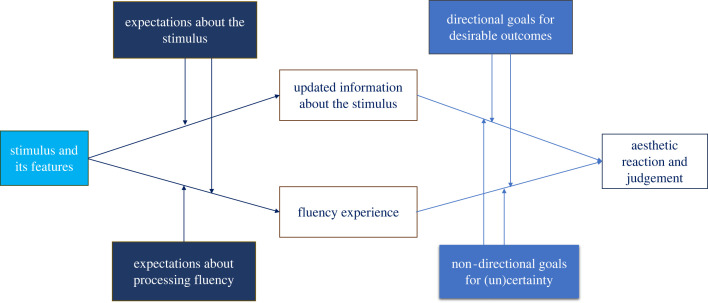
Model that integrates expectations, epistemic goals and fluency.

All these four factors change how fluency enters into aesthetic judgements and what specific impact it has. In short, the current paper offers a substantial revision of the hedonic fluency theory. It also offers a concrete example of benefits derived from the integration of PPF with the EMM. Our new integrated model is shown in simplified form in figure 2, but its detailed assumptions are specified in the text. We hope to show that via this integration we can gain insights into different mechanisms underlying aesthetic judgements within one theoretical model.

## 2. Theory of processing fluency

Processing fluency refers to the subjective experience of ease (or effort) associated with mental processing, such as perceiving, categorizing, recalling, imagining or deciding [13]. The general notion of ‘cognitive experiences' is quite old and goes all the way back to William James, who talked about the feelings of effort, familiarity, rightness, or strangeness as always occurring in the background (at the fringe) of mental activity (see discussion in [14]). It is important here that fluency researchers make a distinction between fluency that is perceptual (related to basic stimulus recognition) versus conceptual (related to semantic elaboration processes) [15] as well as a distinction between fluency that is absolute (objectively fast and easy) versus relative (faster or easier relative to expectations) [16]. A trickier distinction is between fluency that is felt (e.g. fast processing that is subjectively experienced as easy) and unfelt (e.g. fast processing whose ease is not represented in phenomenology) [17,18]. We draw on these distinctions later as they become relevant for our argument.

Systematic empirical work on fluency began in the psychology of memory [5]. It has been long known that, for example, repetition, higher contrast, greater intensity or longer presentation times facilitate stimulus processing, as indicated in faster reaction times [19]. The key early observation was that easier processing can, under the right conditions, elicit an ‘illusion of familiarity’—a sense that a stimulus has been experienced before. In the course of this research, it turned out that many different factors (e.g. duration, contrast, clarity, intensity or priming) can influence fluency, and thus elicit such familiarity illusions [20]. This suggested that there is a unified experience of fluency that might be relevant in a broader set of situations.

Indeed, moving beyond basic recognition memory, researchers noted that fluency acts as a cue to a variety of judgements [21]. Among the first demonstrations were judgements of fame [22] and truth [23,24]. For example, making simple statements more fluent by repeating them or presenting them with a higher contrast led participants to judge them as more true. Later research showed extensions of fluency to judgements of risk [25], trustworthiness [26,27] and many additional dimensions (for review, see [28]).

## 3. Hedonic fluency and aesthetic experience

The Hedonic Marking of Fluency Account [6] was an extension of the original cognitive fluency theory to evaluative judgements. Specifically, it proposed that fluency comes with a positive hedonic marking (tinge), which was speculated to arise because fluency reflects favourable internal states (e.g. low energy expense, computational efficiency, goal progress) and signals favourable states of the world (e.g. familiarity, symmetry, typicality). Note that the original hedonic fluency theory did not explore connections to PPF, which emphasize that organisms seek to build an accurate internal model of the world and thus derive value from predictability—as we discuss shortly.

The original empirical support came from experiments using priming, signal-to-noise ratio, clarity, contrast and duration to facilitate or impede processing of simple picture stimuli and basic liking judgements [29]. The investigations were then broadened to many other stimuli and methods. For example, names, including brand names and stock tickers, that are easier to pronounce are more liked, and more purchased, than disfluent names [30–32]. Importantly, the hedonic fluency effects can be picked up by measures of spontaneous approach–avoidance responses [33] and physiological measures. For example, fluent stimuli increase muscle activity over the zygomaticus major, or the ‘smiling’ region of the face [27,34–37].

The key suggestion that the hedonic fluency theory is applicable to aesthetics came from an observation that many features that make objects attractive are the same features that make objects fluent. To explain the logic of the original version of the theory, we go a bit deeper into three examples (symmetry, repetition, prototypicality). However, note that the original theory also discusses the role of such fluency-enhancing features as simplicity, contrast, clarity, presentation duration and priming.

People generally prefer symmetric to asymmetric targets—in logos [38], designs [39–41] or faces [42]. Interestingly, symmetric targets are more fluent [43,44]. People also like repeated stimuli (i.e. show the mere exposure effect) in studies using paintings, calligraphy, words, faces, brand names and also sounds [45–50], though the effect has important boundary conditions [51]. Critically, repetition also enhances fluency, and its derivative—familiarity [52,53]. Another variable that enhances preference is prototypicality. The rich literature on ‘beauty-in-averageness’ documents that for a variety of categories (faces, birds, watches, consumer products), people prefer prototypical stimuli [54,55]. Fluency is a causal contributor to this preference [56–58]. Importantly, research with real-life stimuli documented that with enough exposure to atypical patterns, a shift in fluency and, consequently, preference occurs [53,59,60]. This highlights the role of perceivers' past history in determining what object will be fluent and preferred.

In short, the original hedonic processing fluency theory, and its extension to art, proposed a relatively simple relationship: fluent processing enhances appreciation because it evokes positive affect, whereas disfluent processing reduces appreciation because it elicits negative affect. Obviously, the original fluency theory never claimed to explain all aesthetic judgements, but it made clear that, all else being equal, more fluent works of art should be appreciated, and gratuitously disfluent art penalized. In fact, in early formulations of this theory, the boundary conditions were discussed rather sparsely. Therefore, it is not surprising that over time empirical findings and conceptual frameworks emerged that challenged this simple proposal.

Next, we review these challenges and preview possible solutions—coming from explicitly incorporating the role of expectations (as elucidated by PPF) and goals (as elucidated by EMM). Note that when the hedonic fluency theory was proposed in 2003 and extended to art in 2004, there was little empirical evidence for how expectations and goals shape the impact of fluency on judgements (aesthetic and otherwise). However, as we discuss next, they both play a key role in determining what is fluent, how fluency is experienced and interpreted, and whether and how it is used in judgements.

## 4. Fluency and the role of predictions

The original version of the theory emphasized that the fluency of a stimulus depends either on its objective properties (symmetry, contrast, duration) or perceivers' history with the stimulus or related stimuli (repetition, priming, prototypicality). Critically, the role of those variables was never explicitly articulated in terms of perceivers' ability to make predictions—hypotheses about what comes next—which is a key concept in PPF. Rather, it was proposed that these variables change how ‘easily’ (quickly and without errors) a stimulus is processed via standard associative mechanisms, such as pre-activation in priming or repetition. Early computational modelling of fluency linked it to monitoring of neural network properties, such as settling time or number of network units changing state [61,62]. The later computational modelling work on hedonic fluency of prototypes used either a global match memory model [59] or a statistical feature learning model [63]. Because these later models essentially compute the degree of match between the stored information and the input, and are Bayesian, they are in principle compatible with PPF [64,65]. However, none of this work explicitly discussed the role of predictions as a key concept for understanding fluency and its hedonic effects.

Some readers may see the contrast between these mechanistic approaches and PPF as a mere difference in terminology or levels of analysis. Or perhaps as a difference between a mechanistic (how) account of psychology and neuroscience and a teleological (why) account of PPF. However, PPF makes empirically verifiable claims that perceivers build a generative model of the world, develop predictions, compare predictions with inputs, assess prediction error and even represent confidence about their own predictions, as elaborated shortly. Accordingly, the key insights from PPF for the fluency theory come from two levels: (i) mechanistic debates about the sources of fluency and affect, and (ii) more general considerations of the functions of fluency and affect. Before expanding on that, it is useful to restate the basic tenets of PPF and then discuss how PPF sees fluency and its hedonic marking.

In general, PPF assumes that the basic goal of human cognitive activity is to minimize future surprise by building, on the basis of observed outcomes, an internal model of the world that accurately predicts sensory states [66]. People revise their models when they are confronted with new and surprising information. Importantly, people also execute actions to obtain more information about the world in order to refine their internal model and minimize surprises in the future. There are two types of activities. Whereas pragmatic actions serve exploitation purposes, epistemic actions serve exploration purposes. This process of choosing what environment to explore is referred to as ‘active inference’ and, ultimately serves the purpose of building the most comprehensive yet simple model of the world.

PPF accounts well for a variety of empirical data, especially in the perceptual domain [9]. It also proposes to explain affective phenomena by assuming that perceivers experience reward from moving towards a state of increased predictability. More specifically, some PPF papers talk about ‘affective charge, which specifies changes in the expected precision of (i.e. confidence in) one's action model’ [67]. That is, positive affect does not simply come from the match of the stimulus with prediction, but from changes in the perceived quality of one's generative model. Similarly, other PPF papers link positive affect to better-than-expected reduction of prediction error [68]. This notion that positive affect arises from better-than-expected reductions in prediction error or increases in confidence about one's predictions will become relevant shortly when we talk about relative fluency as a source of positive affect.

### (a) Fluency in the predictive processing framework

What are the specific implications of the PPF for the original fluency account of aesthetic judgement? To answer this question, we need to discuss the concept of fluency under PPF. In essence, PPF suggests that, for example, seeing a related stimulus (priming), seeing a stimulus multiple times (repetition) or seeing various similar instances of a category (prototypicality) all change priors—perceptual and conceptual expectations for the target. As a consequence of all these manipulations, the target evokes less surprise. As Brielmann and Dayan say in their recent paper on modelling hedonic fluency phenomena: ‘We suggest that stimuli that are likely or predictable under the generative model are those that are processed fluently’ [64, p. 1322]. Consistently, these authors equate greater fluency with a more precise match between predictions and the sensory input.

However, other PPF proposals offer more intricate views of fluency. Brouillet & Friston [69] draw on a distinction between unfelt and felt fluency. Unfelt fluency occurs on a lower, sub-personal level (e.g. basic visual expectations), but it can become subjectively felt when surprising. It is then subjectively represented (felt) on a higher, ‘person’ level. Specifically, Brouillet and Friston say: ‘felt fluency can be read as inferring some fluent processing at lower hierarchical levels while, at the same time, issuing top-down predictions that place priors over the mediation of unfelt fluency; namely, precision at lower levels of the hierarchy’ [69, p. 8]. In other words, in this model, felt fluency is more than a simple match—it is a feeling generated at a higher level of the hierarchy that represents precision of low-level matches. Moreover, whether fluency (or disfluency) is felt depends on the amount of surprise, accounting for why we primarily feel ‘relative fluency’.

What do PPF proposals say about hedonic fluency in aesthetic judgements? In our reading of the literature, there is a diversity of views. Some proposals view aesthetic responses as more directly tied to low prediction error *per se* [70]. Specifically, Brielmann & Dayan [64] say ‘the immediate reward derives from the fluency with which the current stimulus is processed (as in fluency theories) and is quantified as the likelihood of that particular stimulus under the current generative model…’ [64, p. 1323]. As mentioned, other proposals suggest that aesthetic response is tied to a better-than-expected rate of prediction error minimization [68]. In any case, the sense in which fluency was used in the original hedonic fluency theory seems most related to the PPF notion of monitoring the relative quality of processing and prediction accuracy at a high, personal level of hierarchy. We will return to the issue of relative fluency.

### (b) Expectations about stimuli and fluency itself

One important general observation that naturally comes out of PPF, but was not considered in the original fluency theory, concerns the dynamic changes in stimulus fluency as a function of the currently active predictive model in a person's mind. This is because predictions generated at the top level of the processing hierarchy propagate to lower levels, where they are checked against incoming (bottom-up) evidence. Basically, how fluent a stimulus is should not be stable, but should dynamically change as a function of the perceiver's current expectations. Supporting this, our work has highlighted that top-down manipulations determine what specific stimulus becomes fluent and what stimulus becomes disfluent. For example, take a stimulus like an androgynous human face. Note that it can serve as an excellent prototype of a broad category (human beings), but also as an atypical example of narrow gender categories (male versus female). Indeed, when participants' task is simply to detect the presence of faces, the androgynous faces are fluent and liked. However, when participants' task is to categorize faces into male or female, the very same androgynous face becomes disfluent and disliked [71]. Similar dynamic changes in fluency and preference for the same stimulus as a function of top-down manipulations have been demonstrated with other categories, such as living–non-living [72], ethnic categories [73] and specific emotion categories [27,74]. This dynamic dependence of stimulus fluency on top-down task set has been computationally modelled and accounts well for empirical data on changes in fluency and attractiveness of faces from a variety of categories [63]. In short, we propose that whether the same object is fluent, and benefits from the hedonic consequences of fluency, is moderated by the currently active and dynamically changing model of the world.

A much broader implication is that the impact of expectations on fluency could be one reason why factors such as social norms, expertise and reputation all play a role in shaping an aesthetic experience. There is extensive empirical evidence that individual preferences depend on how the majority, or some valued group, behaves or thinks [75]. This notion has been supported with items such as t-shirts [76], songs [77], food [78], faces [79] and even moral choices [80]. Social influence can occur even when the stimulus is fluent and unambiguous [79]. However, it has even more power when the stimulus is uncertain [81]. We propose that one reason this influence occurs is because social norms, in addition to communicating value, create top-down expectations about the stimulus, which change its processing fluency. We are not aware of evidence for this mechanism in the aesthetic domain, but it is documented in the prejudice domain [82]. Note also that when individuals attribute epistemic authority to themselves, they are less likely to be influenced by others. This effect may hold for art experts, whose broad aesthetic values and specific feelings of fluency may be more highly constrained by personal knowledge and domain familiarity, hence less sensitive to others' expectations. Again, this could be empirically tested.

Importantly, people have expectations not only about the stimuli, but also about the fluency itself—as discussed in research on unexpected fluency. The original insight again came from cognitive work on familiarity illusions, as well as follow-up studies on truth and fame. This work established that cognitive effects of fluency manipulations depend not on the absolute level of fluency but rather on its relative level. Specifically, Whittlesea & Williams [16] showed that it is the discrepancy (deviation) in fluency rather than the (absolute) level of fluency that drives familiarity illusions [16]. This means that the stimuli had to feel *more fluent than expected* in order to create a familiarity illusion. This point was extended beyond the memory domain by studies showing similar relative fluency effects on judgements of truth and preferences [83].

The original hedonic fluency account paid little attention to the role of unexpected fluency. It mostly saw expectations as determining attributions of the source of fluency (if fluency is unexpected, it is more likely to belong to the stimulus). Accordingly, the original account claimed that ‘people continue to enjoy prototypical faces, symmetrical patterns, harmonious chords, and high clarity drawings even after they formed fairly accurate processing expectations for these stimuli’ [7, p. 372]. But despite its plausibility, this claim has not been empirically tested. Still, the theoretical basis for this claim was that the original hedonic fluency theory linked pleasure to the intrinsic value of low effort (e.g. reward for efficiency in energy and coding) as well as the heuristic value of fluency (e.g. familiarity is a heuristic cue to benign environments, prototypicality a cue to low deviance) [18]. In other words, in the original hedonic fluency theory, stimuli can be liked simply because they are ‘easy on the mind’.

By contrast, expectations are key for PPF. Note, however, that even within the PPF framework of hedonic fluency, different readings put different emphasis on the notion of relative fluency. As mentioned, one interpretation equates fluency with a precise match between predictions and the sensory input, which indicates smaller prediction errors. In fact, Brielmann and Dayan [64] modelled classic hedonic fluency effects (exposure, complexity and symmetry) using these simplified assumptions, without incorporating the notion of relative fluency. However, other proposals within PPF tie hedonic fluency to perception of relative *changes* in the expected precision of one's model or in the rate of prediction error minimization [68,69]. Regardless of one's preferred reading of PPF, future research should investigate which aesthetic phenomena are grounded in positive affect resulting from such ‘better-than-expected’ reduction in prediction error. Indeed, some works of art may appeal to viewers only because they resolve initial uncertainty or ambiguity. However, there could be works of art which rely on fluency effects, such as familiarity, prototypicality or symmetry, and continue to produce positive affect even when viewers approach them with well-formed and accurate expectations.

Finally, note that being metacognitively surprised by high fluency tells the perceiver that their actual model of the world is ‘better-than-expected’ (which is a good thing) but also that their model of their own mind is inaccurate (which is not so good). This dynamic may not apply on sub-personal, low levels of information processing. However, on a personal, intentional level, we speculate that perceivers who strongly value accurate metacognition (so that they don't like errors in any direction) might be bothered by surprising fluency. As a loose analogy, a driver confident of their ability to correctly estimate commute time might be bothered by arriving earlier than expected (rather than delighting in discovering their superior driving skills). Future research should explore whether focusing perceivers on their own metacognitive accuracy leads to negative affective consequences when they experience such discrepancies. But, as mentioned, this phenomenon is more likely related to one's high-level, general epistemic goals—a topic we turn to in the next section.

In concluding this section, we hope to have convinced the reader that the fluency theory has much to benefit, both conceptually and empirically, from the PPF. PPF offers important, empirically verified or verifiable insights about the role of internal models and expectations about stimulus processing as well as expectations about fluency itself. Note, however, that the standard PPF could be extended by including a broader consideration of goals [12]. This is because beyond building accurate predictive models, people have other epistemic goals that guide their cognitive activities and affective reactions. This brings us to the next set of challenges.

## 5. Fluency and the role of epistemic goals

A big challenge to the original fluency theory is that people sometimes appreciate what is unexpected, novel and complicated. Indeed, studies report that clearly disfluent visual properties (e.g. complex, atypical, novel, ambiguous stimuli) are sometimes judged as aesthetically pleasing [53,60,84–88]. This challenge led to proposals that the hedonic fluency model may be applicable to automatic reactions but not to more deliberate, interest-driven judgements [89]. Note that preference for unresolvable, inherently ambiguous, uncertain stimuli is a challenge for the standard PPF as well [70,90]. By incorporating the motivational framework, we propose that whether fluent or disfluent stimuli are preferred depends on the observer's epistemic goals.

Another challenge is that the specific content of the work matters hugely for aesthetic judgements. For example, in religious art, judgements of beauty are reserved for noble characters (angels, not devils [91]), and in political art for view-supportive content (e.g. social realism). One reason Picasso's ‘Guernica’ (1937) is admired comes from its powerful and universal anti-war message (figure 1), whereas the work of Paul Gauguin is being reconsidered based on the problematic nature of its content [92]. By incorporating the motivational framework, we propose that what content is preferred also depends on the observer's specific epistemic goals and, critically, that these goals may also change the role of fluency. To elaborate on this point, we first need to introduce the EMM and discuss its relationship to PPF.

The EMM is a general model that assumes that people construct new beliefs (on any topic) from prior beliefs by updating them on the basis of new evidence—either because they are passively confronted with it or they made an active effort to obtain it ([10,93] for a popular discussion). Both components are crucial: (i) the strength of the prior belief (e.g. ‘Based on the excellent reviews I read before coming to this new exhibition, I expect all paintings to be skillfully done’), and (ii) the relevance of new evidence—namely, the degree to which it strengthens or weakens prior beliefs (e.g. ‘The first painting I see is amazing; the exhibition might be even better than I thought’). The outcome of this process depends on whether the new evidence is perceived as strong and relevant (versus weak and irrelevant) and whether their prior belief was held with high (versus low) confidence. For example, the prior reviews could be more or less unequivocal and the quality of the first painting could be more or less unmistakable.

The EMM assumes, as does PPF, that the implied change in prior beliefs, given new evidence, quantifies surprise (informational gain) induced by new information [11,12]. Surprise indicates that something occurred that prior beliefs did not predict. Surprise can happen when a person had no clear expectations (e.g. ‘I don't know who the painter is’) and they updated their beliefs in any direction, or when their clear expectations were violated (e.g. ‘I thought it was Cezanne but in fact the painter was Picasso’). It highlights the important role of *prior expectations* (e.g. expertise on the previous style of the artist, reputation of the gallery, reviews of the movie) in creating a sense of surprise. As we discussed earlier, these prior expectations will also change fluency—with expected events being more fluent.

However, according to EMM, the process of integrating the prior hypothesis with new evidence does not in itself result in affective reactions and positive or negative evaluation, because *active epistemic goals* should be taken into account. This idea stems from the assumption that people are not only updating their beliefs, but those beliefs have motivational relevance. Therefore, if updated beliefs are in line with their goals, people feel positive, but if updated beliefs frustrate their goals people feel negative. For example, imagine a person who actively wants to believe in the possibility of finding lasting love, but harbours doubts. So, the person buys a book of poems about love. If the person then reads a poem that effectively conveys that most relationships endure, the person should respond with positive feelings. However, if the poem effectively conveys that most relationships fail, the person will most likely feel negative. Whereas in both cases the person learned something new about love from the poem, their affective reactions are driven by the implications of this new knowledge for their preferred conclusion. Finally, note that these goals can be consciously accessible, as when a person attends an art exhibition to learn about a new artist. However, they can also be triggered implicitly, as when a sense of boredom sparks an interest in exploring new music pieces. According to EMM, there are two general kinds of these motivational influences—non-directional and directional—which we address in the next two sections [94].

### (a) Non-directional goals to seek versus avoid certainty

The first kind of motivational influence is non-directional. It may express a desire to arrive at a confident conclusion regardless of its content*.* However, people may also have the opposite desire, to be surprised and experience a state of uncertainty. Under the first type of non-directional goal, certain and predictable (i.e. fluent) experiences may be valued. But, under the other type of non-directional goal, unexpected and surprising (i.e. disfluent) experiences are valued. For a person motivated by a search for certainty, it does not matter whether the painting is pro-war or pro-peace, the poem is about enduring or fleeting love, the movie ending is good or bad, and whether the song is sad or happy—as long as they can confidently determine its meaning. By contrast, there are situations when uncertainty is preferred. For example, some people do not want to know the ending of a book from the beginning, they prefer a movie without a clear conclusion, they do not want to know the nature of love, and they appreciate an ambiguous artwork that remains puzzling over the years. An artist may prefer to produce uncertain, disfluent works if their goal is to express indeterminacy, confusion, chaos or meaninglessness [95].

Empirical evidence supports this distinction, showing that both situational factors and individual differences modify preferences for predictable versus ambiguous stimuli. For example, Hansen & Topolinski [96] showed that perceivers prefer atypical over typical patterns after inducing an exploratory mindset but the effect disappears in a control condition. This finding fits with reports that positive mood, which promotes exploration, also increases preferences for atypical over typical patterns [97]. Similarly, appreciation of various uncommon objects increases when value is placed on uniqueness and novelty [98]. Valsesia & Schwarz [99] report that consumers with high uniqueness goals prefer disfluency. Similarly, readers have different preferences for spoilers. For some, spoilers are valued because they give readers a more fluent experience while reading the novel, while other readers strongly dislike and avoid spoilers [100–102].

Finally, work on individual differences such as need for certainty and closure shows that these variables can to some extent predict preferences for ambiguous art [103,104]. For example, Silvia *et al*. [105] examined individual variability in sources of interest for abstract visual art [105]. The authors identified two distinct groups of people. The first, larger group showed a stronger correlation between their interest ratings and the evaluation of art as novel and complex. These individuals scored higher on traits associated with novelty seeking, such as sensation seeking, openness to experience and trait curiosity. The second group demonstrated a stronger connection between their interest and their perceived ability to understand the picture. These results fit the idea that achieving certainty versus remaining uncertain can hold different value for different people. Another individual level variable that has been shown to be positively correlated with abstract art preferences and negatively correlated with representational art preferences is sensation seeking, defined as a need for novel, complex and intense stimulation [106,107]. There is also work showing a positive relation between appreciation of non-representational, abstract art and the personality trait of schizotypy, which is associated with openness to unusual experiences [108]. However, the engagement of schizotypal inidividuals with non-representational art may be related less to uncertainty preference than to the propensity of such individuals to extract meaning from, or impose meaning on, abstract or indeterminate patterns [109]. Perhaps the strongest evidence for genuine appreciation of uncertainty comes from research on Semantic Instability experiences, showing that some viewers have positive reactions to intrinsically ambiguous works, even when these viewers cannot eventually settle on an interpretation [2,87].

In short, our revised framework emphasizes the importance of value associated with fluency itself. If a non-directional goal of reducing uncertainty is active, the original fluency theory applies and higher fluency elicits positive affect [7]. However, if the non-directional goal of embracing uncertainty is active, the perceiver will prefer a disfluent piece that remains open to interpretation. This leads to a clear (but untested) prediction that individuals will like ambiguous art (e.g. Magritte, DeChirico, Esher) when their goal is just thinking about it, but they will dislike it if their goal is to form a clear conclusion. Finally, note that all these effects could be qualified by individuals' *a priori* predictions related to processing fluency [110]. This is especially true in the context of aesthetic appreciation within a contemporary museum, because people generally ‘expect the unexpected’ [70]. Highlighting the role of expectations is important because an individual with a strong non-directional goal of uncertainty may be disappointed if the work is too fluent and easily interpretable. The general possibility of ‘disappointment’ by stimuli that do not offer an expected level of visual challenge has support in recent findings by Erle & Topolinski [111].

Interestingly, our account is aligned with what has already been argued by some advocates of the PPF perspective, who point out that people do not solely pursue immediate certainty [70]. As further elaborated by Van de Cruys *et al*. [1], this perspective acknowledges that people sometimes balance their desire for order and closure with a preference for surprise. This occurs because tolerating or even seeking uncertainty offers several long-term advantages. First, when individuals effectively cope with uncertainty before reaching a satisfactory resolution, it boosts their self-efficacy in managing uncertainty, encouraging future exploratory behaviour rather than reinforcing hasty cognitive closure. Second, people might embrace uncertainty because of the pleasure they anticipate experiencing when it is eventually resolved, such as when a reader reaches an intellectually rewarding denouement at the end of a crime story.

Both of these reasons, ultimately related to reducing uncertainty in the long run, lead to the question of whether one can appreciate an artwork even when it does not promise decipherability at the end, or when the expected reduction of uncertainty might be minimal or none, often referred to as ‘mere uncertainty’. There might still be psychological benefits of becoming comfortable with uncertainty as such, as expressed in this quote by the art historian Ernst Gombrich [112, p. 23]: ‘art is an institution to which we turn when we want to feel a shock of surprise. We feel this want because we sense that it is good for us once in a while to receive a healthy jolt. Otherwise we would so easily get stuck in a rut and could no longer adapt to the new demands life is apt to make on us. The biological function of art, in other words, is that of a rehearsal, a training in mental gymnastics which increases our tolerance for the unexpected.’ This is an intriguing possibility, which requires further empirical investigation. Next, we turn to another kind of epistemic goals that shape reactions to art.

### (b) Directional goals to seek desirable (and avoid undesirable) outcomes

The second kind of motivational influence is directional and denotes the desirability or undesirability of specific outcomes that a person wants to achieve or avoid, respectively. For instance, typically people want to be healthy rather than sick, accepted by others rather than ostracized, admired for their achievements rather than met with contempt, or have their ideas implemented rather than discarded. The epistemic nature of these motivations lies in the fact that individuals form beliefs that denote whether those outcomes were achieved or could be achieved. Namely, they want to *believe* that these outcomes occurred or could occur, and when they arrive at such a desired conclusion, they experience positive affect. However, if the beliefs convey an undesirable truth about the outcome they would rather avoid, people experience negative affect.

This kind of motivational influence may seem initially less obvious in the context of art, which is often associated with the primary goal of aesthetic pleasure. However, note there are many other specific goals that can be satisfied or frustrated by art pieces. As mentioned earlier, art can be appreciated based on its content, such as when religious art upholds moral values or political art advocates for ideological goals. For instance, if the painting shows people enjoying fruits of immoral activities such as cheating at cards, viewers with just-world beliefs might not appreciate learning about it even if the craft is superb (e.g. La Tour's ‘The cheat with the ace of clubs’, figure 1). Similarly, prude viewers may dislike learning more about nudes (e.g. recent US debates about the harms of teaching children about Michelangelo's ‘David’). These observations are consistent with empirical evidence showing that learning information about an artist's immoral behaviour in real life influences not only the viewer's moral judgements, but even the very perception and aesthetic evaluation of the artwork [113]. This is in part because there is a stronger connection between artistic works and their creators' personalities and behaviours than in other domains [114], which makes it more difficult to separate the evaluation of art from the unacceptable behaviours of the producers.

Note that directional epistemic goals can sometimes minimize the role, or override the impact of fluency (whatever the impact is). One example is Picasso's widely admired Guernica (1937) where each separate element is difficult to identify, leading to a general experience of ‘disfluency' (figure 1). According to the original fluency theory, this should cause a negative reaction to this challenging piece. Similarly, certainty-seeking viewers should dislike it because of its ambiguity. It may be argued that people admire Guernica because many viewers these days are certainty-avoiding and thus value disfluency. However, it seems clear that viewers value Guernica for other reasons, and that non-directional goals or disfluency simply matter little. The admiration for the piece—even among those struggling to decipher it—stems at least partly from its compelling anti-war message, which is congruent with directional epistemic goals (desirable conclusions) held by the viewers. In short, here the content that endorses and strengthens viewers' anti-war beliefs matters more than processing effort, perhaps leading viewers to ignore (dis)fluency altogether.

Nevertheless, the ease or difficulty with which viewers can decipher individual elements of the piece may still change the impact of the content. However, this impact is not straightforward. Sometimes perceivers engage more deeply with specific content when it is *disfluent* [95,115]. For example, noticing a seemingly incongruent lightbulb in ‘Guernica’ may lead the viewer to discover its deeper meaning. Also, sometimes greater fluency facilitates access to *negative* meaning [58,116]. For example, greater ease of recognizing the grieving mother in ‘Guernica’ may help the viewer to better decipher the antiwar message. But it may also lead the viewer to be more upset by the horror of the event. The possibility that greater fluency can sometimes *enhance* negative reactions was only peripherally mentioned in the original fluency theory. Yet, it is key for frameworks like the Fluency Amplification Model, which proposes that fluency can enhance access to both positive and negative content [116]. Indeed, recent work has shown that *greater* ease in recognizing a pattern can lead to more *negative* ratings of that pattern, if the pattern belongs to a negative category [58]. There is a debate as to whether this greater ease of access to negative content can simultaneously coexist (and combine) with the positivity deriving from ease, or whether the positive and negative effects of fluency are symmetrical [58,116], but for now, we simply acknowledge these complex interactions.

### (c) Interplay between epistemic goals

Given that most of the time people pursue multiple goals, both directional and non-directional, the final impact on aesthetic judgement emerges from the interplay between these two types of epistemic goals. The relative strength of directional versus non-directional goals should determine the extent to which fluency (resulting from factors such as novelty, ambiguity or surprisingness) impacts aesthetic judgements. On some occasions, non-directional goals will have greater relative strength. On other occasions, however, specific directional goals will be stronger and may overcome, or even override, considerations of fluency, as we discussed in the case of ‘Guernica’. As a consequence, a preference may emerge for art that lacks fluency, or a rejection of art despite its high fluency. This phenomenon arises when the alternative goals served by these artworks are deemed either desirable or undesirable. For instance, people might dismiss otherwise excellent art if it is incompatible with their more crucial directional goals (e.g. Michelangelo's ‘David’ for viewers who have moral concerns with nudity). On the other hand, people may appreciate even poor art if it fulfils other goals. For instance, people appreciate clumsy art made by their children because it meets family related goals.

Interestingly, people may form multiple judgements of the same piece of art based on different pieces of information and different goals—a point emphasized by integrative models of aesthetic judgements [117]. An exemplar of such a scenario could be the works of Leni Riefenstahl, a filmmaker whose movies served as propaganda for Hitler's regime. Here, the activation of multiple contradictory goals can generate inconsistent reactions even among sophisticated art critics. Consider Susan Sontag's stance as an example. At one point she championed Leni Riefenstahl's movies, asserting their status as outstanding art despite their despicable content. [‘…we can, in good conscience, cherish works of art which, considered in terms of ‘content,’ are morally objectionable to us…. to call Leni Riefenstahl's ‘The Triumph of the Will’ and ‘The Olympiad’ masterpieces is not to gloss over Nazi propaganda with aesthetic lenience. The Nazi propaganda is there, too, which we reject at our loss. (…) Through Riefenstahl's genius as a filmmaker, the ‘content’ has—let us even assume, against her intentions—come to play a purely formal role.’] Yet, in an essay Sontag wrote in 1975, ‘Fascinating Fascism’, she underwent a complete reversal of her position and opposed the rehabilitation of an artist who actively and willingly served a totalitarian regime. Future research could investigate both the underpinnings and consequences of cases where directional and non-directional goals clash for aesthetic judgements.

Another fascinating question pertains to the influence of directional goals on the experience of fluency itself. For instance, if an individual encounters the same artwork, but its meaning either aligns with or contradicts their directional goals, does this not only impact their overall evaluation of the artwork but also affect how they experience it in terms of fluency? Let us consider again the scenario where the ideological message conveyed by a particular piece of art resonates with or contradicts a person's cherished social values. Could these factors potentially enhance or diminish the perceived ease with which that artwork is processed? An admirer of social realism may find those pieces easy to process, but also a detractor well familiar with the tropes. Although this hypothesis has yet to be tested in empirical studies, it presents a promising avenue for future explorations. Investigating whether alignment or contradiction with directional goals influences the actual fluency and appreciation of art could provide insights into the mechanism of aesthetic experiences.

## 6. Conclusion

To conclude, the goal of this paper was to revise and extend the original fluency account of aesthetic judgement by incorporating insights from the PPF and the EMM. Given that an integration of these two theoretical perspectives is currently debated in discussions about the general nature of cognition [93], it should be explored in the domain of aesthetics. Our proposed model is depicted in figure 2, which illustrates the key factors and suggests how they may enter into the processing chain.

In sum, we proposed that four factors should be incorporated into the fluency account. The first are perceivers' expectations about the stimulus—because they change stimulus fluency. The second is perceivers' expectations about fluency itself—because aesthetic experiences can arise from surprising fluency. The third factor is the value associated with fluency and certainty itself—reflecting perceivers' non-directional epistemic goals. Sometimes fluency is valued but at other times disfluency is appreciated, especially in modern art. The fourth factor is the perceiver's specific directional epistemic goals when processing the stimulus. After all, perceivers have preferences about acquiring specific knowledge, reaching a specific conclusion, or even experiencing a specific feeling (e.g. discomfort, elevation). These goals make participants value certain content. Critically, these specific goals may also change whether and how fluency plays a role. We hope that future theoretical developments and empirical research on fluency in aesthetics will lead to further refinements of this framework. For a list of some open questions and study ideas suggested here, please see Appendix A.

## Data Availability

This article has no additional data.

## Appendix A: Some open questions and suggested studies

1. Why do people enjoy fluent stimuli when they are unsurprising? Or do people only get hedonic benefits from fluency when it is better-than-expected? In what settings and for what stimuli? Future studies could address these questions by measuring and manipulating actual fluency and fluency expectations.
2. Are people ever upset by incorrectly predicting their fluency? Even if fluency is better-than-expected? For example, when they grasp a meaning of an artwork or solve a visual puzzle faster than expected? If so, does the upset result from less time to enjoy the piece? Or is it because they were metacognitively wrong? Are people upset only by adverse variance in prediction (worse than expected) and never bothered by advantageous variance (doing better than expected)? What about people who strongly value accurate metacognition? These people may not like errors in any direction.
3. How are perceivers' expectations and the resulting fluency influenced by others? What about art experts, whose expectations come to a larger extent from their personal knowledge of art?
4. Our new framework emphasizes the importance of considering the value associated with fluency itself. If a non-directional goal of reducing uncertainty is active, the original fluency theory should apply, and higher fluency should elicit positive affect. However, if the non-directional goal of embracing uncertainty is active, the perceiver should prefer a disfluent piece that is always open to interpretation. This predicts that individuals will like ambiguous art (e.g. Magritte, DeChirico, Esher) when their goal is just thinking about it, but they will like it less if their goal is to form a clear conclusion. Would an individual with a strong non-directional goal of uncertainty be disappointed if the work is too fluent and interpretable?
5. Do people ever appreciate an artwork even when they do not expect it to be decipherable and they know for sure that the reduction of uncertainty might be minimal or none? If so, why do they appreciate and engage with it?
6. People form complex judgements of the same piece of art based on different goals. Research could investigate cases where non-directional and directional goals match or clash and test the implications for aesthetic judgements. For example, participants could see artwork that generates pleasure from resolving ambiguity. In addition, this artwork would convey meaning that is consistent or inconsistent with their goals (e.g. the ambiguous work resolves into an anti-war or pro-war symbol). How would, say, pacifist participants integrate low-level visual pleasure with feelings resulting from consistency or discrepancy with their high-level goals?
7. Related to this, how do these high-level goals influence the very experience of fluency and its value? Could consistent social (e.g. religious) goals enhance the ease with which the religious artwork is processed? An admirer who is a believer may find those pieces easy to process, but also a critic well familiar with and tired of the tropes. What kind of fluency would an atheist experience in such a situation? Would an atheist prefer a religious work that is disfluent?
8. How do directional and non-directional goals impact the judgements and reactions of art experts compared to novices? When do experts care more about particular content of an artwork, and when do they care more about fluency?
